# Enhanced Mechanical Properties and Degradation Control of Poly(Lactic) Acid/Hydroxyapatite/Reduced Graphene Oxide Composites for Advanced Bone Tissue Engineering Application

**DOI:** 10.3390/biomimetics9110651

**Published:** 2024-10-23

**Authors:** Francis T. Omigbodun, Bankole I. Oladapo

**Affiliations:** 1 Wolfson School of Mechanical, Electrical and Manufacturing Engineering, Loughborough University, Loughborough LE11 3TU, UK; 2 The Manufacturing Technology Centre, Coventry CV7 9JU, UK; 3 School of Science and Engineering, University of Dundee, Dundee DD1 4HN, UK; boladapo001@dundee.ac.uk

**Keywords:** biodegradable composites, bone tissue engineering, tissue regeneration, composite scaffolds, nanocomposites, additive manufacturing

## Abstract

This study explores the enhancement of poly(lactic acid) (PLA) matrix using calcium hydroxyapatite (cHAP) and reduced graphene oxide (rGO) for developing composite scaffolds aimed at bone regeneration applications. The PLA composites were fabricated through solvent evaporation and melt extrusion and characterized by various techniques, including thermogravimetric analysis (TGA), differential scanning calorimetry (DSC), Fourier transform infrared spectroscopy (FTIR), scanning electron microscopy (SEM), and mechanical testing. The incorporation of cHAP and rGO significantly improved the thermal, mechanical, and morphological properties of the PLA matrix. Mechanical testing revealed that adding 10% cHAP and varying amounts of rGO (0.1%, 0.3%, 0.5%) enhanced tensile and compressive strengths, with the highest improvements observed at 0.5% rGO content. Thermal analysis showed increased thermal stability with higher degradation temperatures for the composites. Spectroscopic analyses confirmed the effective integration of cHAP and rGO into the PLA matrix with characteristic peaks of the fillers identified in the composite spectra. In vitro, degraded action tests in phosphate-buffered saline (PBS) at pH 7.4 over 12 months indicated that composites with higher rGO content exhibited lower mass loss and better mechanical stability. Furthermore, finite element analysis (FEA) simulations were performed to validate the experimental results, demonstrating a strong correlation between simulated and experimental compressive strengths. This novel approach demonstrates the potential of PLA/cHAP/rGO composites in creating effective and biocompatible scaffolds for tissue engineering, providing a comprehensive analysis of the synergistic effects of cHAP and rGO on the PLA matrix and offering a promising material for bone regeneration applications.

## 1. Introduction

The ongoing challenge in bone tissue engineering (BTE) is to develop scaffolds that support bone regeneration and exhibit the necessary mechanical strength, bioactivity, and degradation control. While considered the gold standard for bone repair, traditional bone grafts, such as autografts and allografts, suffer from significant limitations. Autografts are limited by donor site morbidity and supply constraints, while allografts pose risks of infection and immune rejection, which restrict their widespread use [1]. Consequently, a concerted effort has been made to develop synthetic, biodegradable materials to overcome these challenges.

Among synthetic polymers, poly (lactic acid) (PLA) has gained considerable attention due to its biocompatibility, biodegradability, and ease of fabrication [2]. PLA has been extensively used in biomedical applications, including scaffolds for tissue engineering, but its use in bone tissue engineering remains limited due to several critical drawbacks. Most notably, PLA suffers from inadequate mechanical strength and bioactivity, making it unsuitable for load-bearing applications in bone regeneration [3]. These shortcomings have prompted researchers to explore strategies to reinforce PLA and improve its performance.

Incorporating bioactive fillers into the PLA matrix has been one of the primary strategies to enhance its mechanical properties and bioactivity. Calcium hydroxyapatite (cHAP), a naturally occurring mineral in bone, is one of the most studied fillers due to its osteoconductive properties and ability to integrate with bone tissue [2,4]. The addition of cHAP into PLA matrices has shown potential in improving the stiffness and bioactivity of the composite material, making it a more viable candidate for bone regeneration. However, while cHAP enhances bioactivity, its impact on the overall mechanical properties is often insufficient to meet the demands of load-bearing bone tissue engineering applications.

In parallel, integrating nanomaterials like reduced graphene oxide (rGO) has garnered attention for its potential to dramatically enhance polymer-based composites’ mechanical, electrical, and thermal properties [5]. rGO’s exceptional mechanical robustness, high surface area, and electrical conductivity make it an ideal candidate for reinforcing polymer matrices. Previous studies have shown that including graphene-based materials in polymers can significantly improve their tensile and compressive strengths, thermal stability, and degradation resistance [6]. However, using rGO in combination with cHAP in PLA scaffolds remains relatively unexplored, particularly concerning its long-term degradation behavior and the synergistic effects of these fillers on the PLA matrix.

The novelty of this study lies in the simultaneous incorporation of cHAP and rGO into the PLA matrix to create a composite scaffold with superior mechanical properties, enhanced bioactivity, and controlled degradation behavior tailored specifically for bone tissue engineering applications. While the individual effects of cHAP and rGO on polymer matrices have been explored in isolation, this study uniquely combines these fillers to investigate their combined impact on PLA composites. The hypothesis is that cHAP will enhance the osteoconductivity and bioactivity of the composite. At the same time, rGO will provide the necessary mechanical reinforcement and thermal stability, creating a scaffold with properties superior to those of either filler alone.

This study aims to address several critical issues identified in the field of bone tissue engineering:

1. **Mechanical Strength**: PLA, on its own, lacks the mechanical strength required for load-bearing applications. By incorporating varying concentrations of rGO (0.1%, 0.3%, and 0.5%) and cHAP (10%), this study seeks to significantly improve the tensile and compressive strengths of PLA, making it more suitable for bone tissue engineering [7]. The mechanical properties of the composites are tested using tensile and compression tests to determine the optimal rGO concentration for achieving the highest mechanical performance.
2. **Thermal Stability**: Another limitation of PLA is its relatively low thermal stability, which can affect its performance in physiological conditions. The inclusion of rGO, known for its high thermal conductivity and strength, is expected to enhance the thermal properties of the PLA composite, thereby improving its resistance to thermal degradation [8]. This study investigates the thermal properties of the composite using thermogravimetric analysis (TGA) and differential scanning calorimetry (DSC).
3. **Bioactivity and Osteoconductivity**: cHAP has long been recognized for its osteoconductive properties, promoting bone growth and integration. By incorporating cHAP into the PLA matrix, this study aims to enhance the bioactivity of the composite, making it more conducive to bone tissue regeneration. The degree of cHAP dispersion and interaction with the PLA matrix is analyzed through scanning electron microscopy (SEM) and Fourier transform infrared spectroscopy (FTIR) to ensure effective incorporation and bioactivity [9].
4. **Degradation Control**: For bone tissue engineering, scaffolds must exhibit controlled degradation rates aligning with the new bone formation rate. Pure PLA often degrades too quickly or unevenly, compromising scaffold integrity. The introduction of rGO is expected to slow the degradation rate, ensuring the scaffold maintains its mechanical integrity over time. This study investigates the degradation behavior of the composites through in vitro degradation tests conducted in phosphate-buffered saline (PBS) at pH 7.4, simulating physiological conditions over 12 months [10].
5. **Finite Element Analysis (FEA) for Performance Prediction**: In addition to experimental characterization, this study employs finite element analysis (FEA) simulations to model the mechanical performance of the scaffolds under physiological conditions. FEA allows for predicting stress distribution and failure points within the scaffolds, offering insights into their suitability for load-bearing applications. This study’s use of computational models provides a comprehensive understanding of how the scaffolds will perform in real-life scenarios [11,12].

This study contributes to developing next-generation biomaterials for bone tissue engineering by addressing these issues. The PLA/cHAP/rGO composite scaffolds developed in this research represent a novel approach to improving the mechanical, thermal, and degradation properties of PLA-based scaffolds, providing a promising material for bone regeneration applications. The findings from this study are expected to advance the field of biomaterials and offer new insights into the design and fabrication of scaffolds for bone tissue engineering.

## 2. Materials and Methods

### 2.1. Materials

#### 2.1.1. Poly(Lactic Acid)

The PLA used (poly(L-lactic acid)) in this study was Ingeo™ BiopPolylacticD, sourced from Nature Works LLC, Plymouth, MN, USA. This grade of PLA is known for its high molecular weight, good mechanical properties, and biodegradability, making it suitable for biomedical applications. PLA 4060D has a density of approximately 1.24 g/cm^3^ and a melting temperature of around 150–160 °C. It is supplied in pellet form and is known for its high clarity and low residual monomers.

#### 2.1.2. Reduced Graphene Oxide (rGO)

The rGO was acquired as graphene nanoplatelets from Graphene supermarket. rGO was chosen because of its superior mechanical strength, electrical conductivity, and biocompatibility [5]. The specific surface area and dispersion quality of rGO are critical for enhancing the properties of the PLA composite. The rGO nanoplatelets typically have a thickness of a few nanometers and a lateral size of several micrometers, with a few manometers of thickness in the range of 400–700 m^2^/g.

#### 2.1.3. Calcium Hydroxyapatite (cHAP)

The cHAP was provided by HIMED Himteco Medical Applications Inc., Bethpage, NY, USA. cHAP is a bioceramic material that mimics the mineral composition of bone, promoting osteoconductivity and biocompatibility. Its particle size and purity influence the composite’s bioactivity and mechanical properties. Influences a particle size distribution with an average diameter of 50–200 nm, high purity (>99%), and a Ca/P ratio of approximately 1.67, closely resembling natural bone mineral.

#### 2.1.4. Chloroform

The chloroform of analytical grade was sourced from JT Baker, Phillipsburg, NJ, USA. Chloroform was used as a solvent to prepare solutions for the solvent evaporation method. Its volatility and ability to disappear are essential for producing a homogenous polymer solution. Chloroform is a volatile organic compound with a boiling point of 61.2 °C. Handling chloroform in a well-ventilated area and using appropriate personal protective equipment to handle health hazards is essential.

These materials were selected to synergistically improve the critical PLA matrix’s mechanical, thermal, and bioactive properties, aiming to develop a composite material suitable for bone tissue engineering applications.

### 2.2. Composite Preparation

#### 2.2.1. Solvent Evaporation Method

PLA was dissolved in chloroform at a concentration of 10 wt%. The solution was stirred continuously at room temperature until the PLA was disbanded entirely. cHAP and rGO were added to the PLA solution in specified ratios (e.g., 10% cHAP and varying rGO wholly dissolved%, 0.5%). The mixture was stirred for an additional 2 h to ensure uniform dispersion. The mixture was subjected to ultrasonication for 30 min to break up agglomerates and ensure a homogenous distribution of cHAP and rGO within the PLA matrix. The solution was poured into a Petri dish and left to dry under ambient conditions for 24 h, followed by vacuum drying at 50 °C for 12 h to remove residual solvent. The dried film was cut into small pieces and extruded at 180 °C using a twin-screw extruder to form filaments.

#### 2.2.2. Melt Extrusion Method

PLA pellets were dry-mixed with the required amounts of cHAP and rGO. The mixture was fed into a twin-screw extruder and processed at a temperature range of 180–200 °C. The extruded filament was cooled in air and pelletized. The pelletized composite was re-extruded at 180 °C to obtain uniform filaments suitable for 3D printing.

#### 2.2.3. Three-Dimensional Printing of Scaffolds

The composite filaments were loaded into a fused deposition modeling (FDM) 3D printer. The printer settings were optimized with a nozzle temperature of 200 °C, bed temperature of 60 °C, and printing speed of 50 mm/s. Gyroid and Schwartz primitive structures were selected for scaffold designs due to their favorable mechanical properties and porosity. The scaffolds were printed layer by layer with a layer height of 0.2 mm and an infill density of 60%.

### 2.3. Characterization

#### 2.3.1. Morphological Analysis

Scanning Electron Microscopy (SEM) from JOEL UK limited, Hertfordshire, UK, was used to analyze the surface morphology and dispersion of cHAP and rGO within the PLA matrix. Samples were sputter-coated with gold before imaging.

#### 2.3.2. Mechanical Testing

The mechanical properties of the PLA/cHAP/rGO composites were evaluated to ensure their suitability for use as scaffolds in tissue engineering, which requires sufficient mechanical stability to withstand in vivo stresses and mimic the mechanical properties of surrounding tissue. This section compares the tensile and compressive test results with relevant literature.

##### Tensile Tests

Tensile tests were performed using five samples of each material: virgin PLA, PLA dissolved in chloroform and dried, PLA with 10% cHAP, PLA with 10% cHAP and 0.1% rGO, PLA with 10% cHAP and 0.3% rGO, and PLA with 10% cHAP and 0.5% rGO. The samples had nominal dimensions of 70 × 10 mm and were tested using an Instron EMIC DL3000 machine, from Norwood, MA, USA, with a 1 KN load cell at a constant head speed of 5 mm/min, following ASTM D638-14 standards [13].

To calculate the tensile strength (σ) and Young’s modulus (E), the following equations were used:

(1) $$\sigma =\frac{F}{A}$$

where F is the force applied, and AA is the cross-sectional area of the sample.

(2) $$E=\frac{\sigma }{\epsilon }$$

where ϵ is the strain.

##### Compression Tests

The compressive strength of the PLA/cHAP/rGO composites was evaluated using five replicas of each composite, with dimensions of 20 × 20 × 30 mm. The tests were carried out using a Zwick Roell Z0.5 testing machine from Worcester, UK at a head speed of 1 mm/min. The compression modulus was determined from the linear segment of the stress–strain curve and the initial cross-sectional area.

The compressive strength (σ) was calculated as:

(3) $$F=\frac{P}{A}$$

where F is the measured force, and A is the cross-sectional area. Deformation (ϵ) was defined as:

(4) $$\epsilon =\frac{∆h}{h_{o}}$$

where Δh is the height difference at each test point, and h_0_ is the initial height of the specimen.

#### 2.3.3. Thermal Analysis

Thermogravimetric Analysis (TGA) assessed the composites’ thermal stability and degradation temperatures. Measurements were carried out from room temperature to 600 °C at a heating rate of 10 °C/min under a nitrogen atmosphere. Differential Scanning Calorimetry (DSC) measured the thermal transitions such as melting temperature (T_m_) and glass transition temperature (T_g_). Samples were heated from −20 °C to 200 °C at 10 °C/min.

To determine the weight loss (WL) during TGA, the following equation was used:

(5) $$\mathrm{WL}\;(\%)=\frac{W_{i}-W_{f}}{W_{i}}\times 100$$

W_i_ is the initial weight, and W_f_ is the final weight after heating.

#### 2.3.4. Spectroscopic Analysis

Fourier transform infrared spectroscopy (FTIR) identified the chemical bonds and interactions between PLA, cHAP, and rGO. Spectra were recorded from 4000 to 400 cm^−1^ using the KBr pellet method.

### 2.4. In Vitro Degradation

Sample preparation involved scaffold samples with varying rGO content (0.1%, 0.3%, 0.5%) and control samples (pure PLA and PLA-cHAP). Samples were immersed in phosphate-buffered saline (PBS) at pH 7.4 to simulate physiological conditions. The samples were incubated at 37 °C for up to 12 months. At predefined intervals (1, 2, 3, 6, 9, and 12 months), samples were removed, dried, and tested for mass loss, calculated as the weight loss percentage. Water absorption was measured as the percentage of water uptake. Tensile and compressive tests were performed to evaluate the degradation effects on mechanical strength.

These detailed materials and methods provide a comprehensive overview of the experimental procedures used in this study.

To calculate the mass loss (ML) during in vitro degradation, the following equation was used:

(6) $$\mathrm{ML}\;(\%)=\frac{W_{initial}-W_{final}}{W_{initial}}\times 100$$

where $W_{initial}$ is the initial weight and $W_{final}$ is the weight after degradation.

Water absorption (WA) was calculated using:

(7) $$\mathrm{WA}\;(\%)=\frac{W_{wet}-W_{dry}}{W_{wet}}\times 100$$

in which $W_{wet}$ is the weight of the sample after immersion in PBS and $W_{dry}$ is the initial dry weight.

### 2.5. Simulation Studies

Finite element analysis (FEA) simulations were conducted to validate the experimental compressive strengths of the PLA/cHAP/rGO composite scaffolds, focusing on two intricate lattice structures: Gyroid and Schwartz Primitive. The simulations aimed to replicate the mechanical conditions applied during experimental testing to provide a reliable prediction of the mechanical performance of these scaffolds.

#### 2.5.1. Design of Specimens with Lattice Structure

The simulation models were developed using SpaceClaim from the ANSYS software suite 2023b. To match the experimental specimens, the lattice structures were designed with 20 mm diameter and 30 mm height cylindrical geometries. The design included 20% relative density to maintain structural integrity and facilitate efficient simulations.

#### 2.5.2. Material Property Assignment

Material properties were assigned based on the experimental data, including Young’s modulus, Poisson’s ratio, and density. The values for PLA, cHAP, and rGO were derived from mechanical testing and the literature. The following properties were used:

These properties were used to create composite material models with varying rGO content (0.1%, 0.3%, 0.5%).

The relative density *ρ* and the Poisson’s ratio ϑ are then calculated from Equations (8) and (9).

The modulus of elasticity [E] is retrieved from the experimental data in this research.

(8) $$\rho_{c}=\frac{\rho \;PLA\;V\;PLA+\rho \;cHAp\;V\;cHAp+\rho \;rGO\;VrGO}{V\;PLA+V\;cHAp+VrGO}$$

ϑ_*c*_ = ϑ _PLA_*V*_PLA_ + ϑ _*cHAp*_
*V*_*cHAp*_ + ϑ _*rGO*_
*V*_*rGO*_ (9)

The modulus of elasticity [E] for the rGO is 1000 GPa, PLA is 3.42 GPa, and cHAp is 10 GPa [14,15]. The poison ratio for PLA is 0.35, cHAP is 0.30, and rGO is 0.33, from which another composite poison ratio is derived. The relative density (*ρ*) of PLA is 1.310 kg/m^3^, cHAp *ρ* used is 3150 kg/m^3^, and *ρ* of rGO is 1955 kg/m^3^ [13]. PLA’s rigidity or shear modulus (G) modulus is 2.6 Gpa, cHAp is 3.943 GPa, and rGO is 427.35 GP.

From Equations (10) and (11), 89.9% of PLA, 10% of cHAp, and 0.1% of rGO give the expression:

*ρ*_*c*_ = 1310 kg/m^3^ × 0.899+ 3150 kg/m^3^ × 0.1 + 1955 kg/m^3^ × 1 × 10^−3^ (10)

*ρ*_*c*_ = (1178 + 315 + 1.955) kg/m^3^ = 1494.95 kg/m^3^ (11)

The same steps were used in calculating the poison ratio and the relative density of the remaining compositions, as outlined in Table 1 above.

#### 2.5.3. Mesh Generation

A tetrahedral meshing approach was employed to capture the intricate lattice geometries accurately. Mesh refinement was applied selectively to critical areas to ensure satisfactory detail capture while maintaining computational efficiency.

#### 2.5.4. Boundary Conditions and Loading

The base of the scaffold models was fixed to simulate the support conditions during experimental compression tests. A compressive load was applied to the top of the structures to replicate the experimental loading conditions. The displacement boundary conditions were set to allow the model to deform naturally under the applied load.

#### 2.5.5. Finite Element Analysis

The FEA simulations evaluated the stress distribution and identified the maximum von Mises stress within the lattice structures. The simulations provided detailed insights into the mechanical behavior under compressive loading.

## 3. Results and Discussion

### 3.1. Visual and Dimensional Aspects of the Scaffolds of PLA/cHAP/rGO Composites

**Figure 1:**
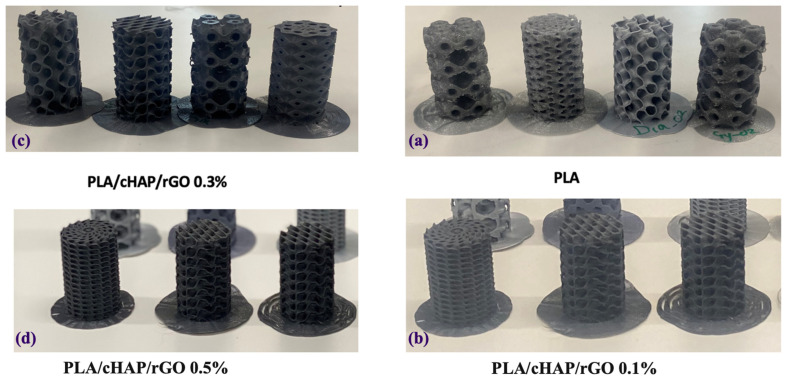
Stereoscopic micrographs of PLA/cHAP/rGO gyroid and Schwartz primitive scaffolds of (**a**) PLA, (**b**) PLA/cHAP/rGO 0.1%, (**c**) PLA/cHAP/rGO 0.3%, and (**d**) PLA/cHAP/rGO 0.5% obtained by 3D printing on an FDM printer (top view).

The first point of analysis observed is that the distinction between the scaffolds’ ordinary PLA and the introduction of reduced graphene oxide is perceived by its coloration. The composite scaffolds have a darker color compared to ordinary PLA. Reduced graphene oxide is black and affects the physical appearance of the printed composite scaffold in terms of coloration. Another point of attention is that the additive manufacturing process using PLA eventually left residues of extruded material between the printing layers, internally between the projected pores, due to the retraction at the time of deposition and movement of the print nozzle. However, such residues may have no direct impact on the objectives of this study and will be verified in greater detail with the use of SEM. Figure 1 presents the visual aspects of the PLA/cHAP/rGO scaffolds (gyroid and Schwartz primitive): PLA/cHAP/rGO 0.1%, PLA/cHAP/rGO 0.3%, and PLA/cHAP/rGO 0.5%.

### 3.2. Morphological Analysis

**Figure 2:**
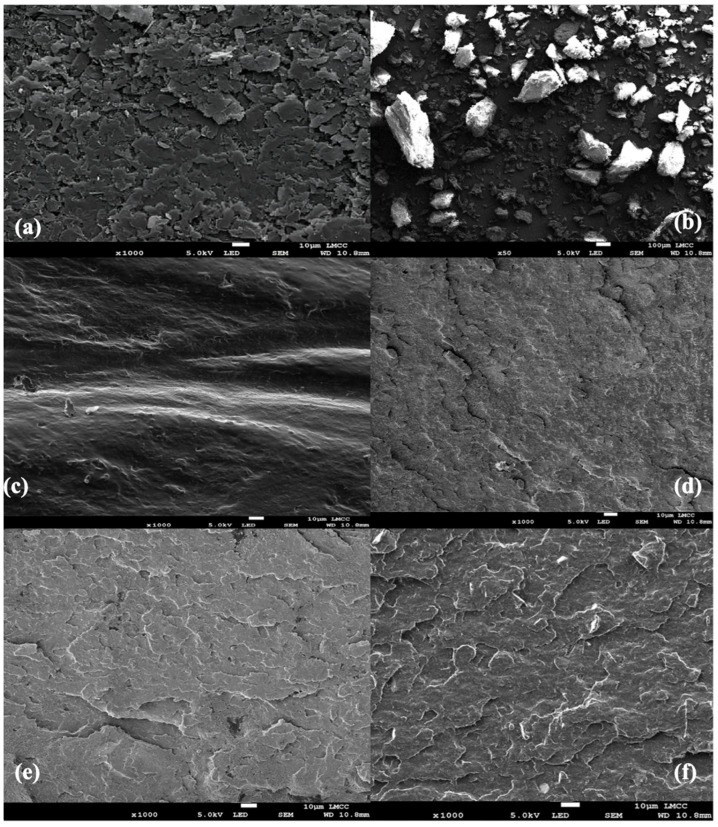
SEM images of (**a**) pure PLA, (**b**) cHAP, (**c**) rGO, (**d**) PLA/cHAP/rGO 0.1% (**e**) PLA/cHAP/rGO 0.3% (**f**) PLA/cHAP/rGO 0.5% composites, showing the changes in surface morphology with the addition of cHAP and rGO and incorporating cHAP and rGO results in a rougher surface compared to pure PLA, indicating successful dispersion of the nanofillers—scale bar: 10 µm.

Scanning Electron Microscopy (SEM) images revealed the successful incorporation of calcium hydroxyapatite (cHAP) and reduced graphene oxide (rGO) into the PLA matrix. The composites exhibited a rougher surface than pure PLA, indicating good nanofiller dispersion [16,17]. The presence of rGO was confirmed by its distinct layered structure and increased surface roughness. Similar findings were reported by Gomez et al. [16], who observed enhanced surface roughness in PLA composites with the addition of graphene oxide. Several samples, including rGO, cHAP, PLA, and PLA/cHAP/rGO composites, are shown in the SEM images. Figure 2a demonstrates that the surface of rGO is not smooth and that rGO has a layered structure. Wrinkles can be seen on the rGO sheets as they are stacked, indicating the characteristic morphology of rGO nanosheets [18]. The disorganized, wrinkled structure of the rGO nanosheets is likely due to the solvent evaporation technique used during the composite preparation process, which affects the force distribution within the rGO layers [19]. Figure 2b shows that cHAP tends to cluster due to the chemical precipitation technique used for its synthesis [20]. The clustered appearance of cHAP particles is evident, which influences the overall surface morphology of the composites [21]. Figure 2c shows that PLA has a relatively flat and smooth surface, the baseline for comparing the modified composites [22].

Figure 2d–f illustrate that the surfaces of PLA/cHAP/rGO composites are rougher than the unmodified PLA. The composites exhibit joints and lumps, indicating that rGO and cHAP nanoparticles have successfully bonded to the PLA surface. The rough surface morphology suggests good dispersion and interaction between the PLA matrix and the nanofillers. The surface of rGO nanosheets in the composites appears more disorganized, with many tangles attributed to the solvent evaporation technique disrupting the force distribution within the rGO layers. Additionally, in the cHAP/rGO composite, cHAP particles remained attached to the surface of the rGO nanosheets after processing, indicating a strong solid interaction between cHAP and rGO.

**Figure 3:**
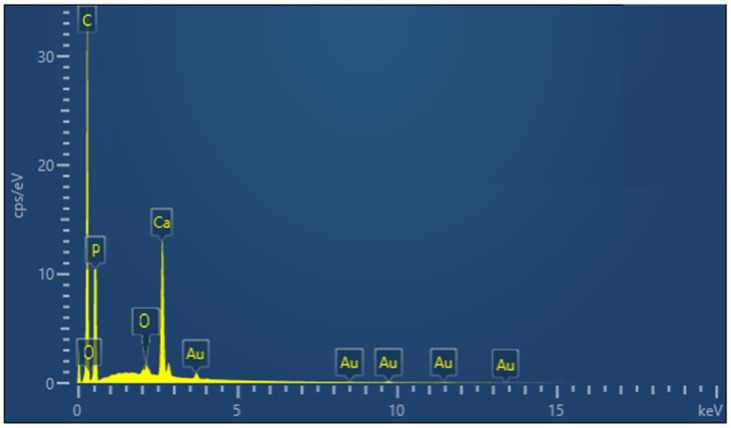
EDS spectrum of PLA/cHAP/rGO 0.5% sample.

Energy Dispersive Spectroscopy (EDS) spectra of the PLA/cHAP/rGO 0.5% samples (Figure 3) confirmed critical vital elements’ presence. The EDS spectra identify elements such as carbon (C), oxygen (O), phosphorus (P), calcium (Ca), and gold (Au). The presence of C, O, P, and Ca confirms the incorporation of cHAP and rGO in the PLA/cHAP/rGO composite. The Au element is attributed to the gold plating used in the SEM analysis to improve the conductivity of the sample.

### 3.3. Mechanical Properties

The mechanical properties of the PLA/cHAP/rGO composites were evaluated to ensure their suitability as scaffolds in tissue engineering, which requires sufficient mechanical stability to withstand in vivo stresses and mimic the mechanical properties of surrounding tissue [23,24]. This section compares the tensile and compressive test results with relevant literature.

The support structures used in tissue engineering must provide an environment with mechanical properties similar to surrounding tissue. Mechanical characterization is crucial to determining the suitability of materials for these applications [25]. The mechanical properties were assessed through tensile and compressive tests to evaluate the effects of cHAP and rGO additives on the PLA matrix.

#### 3.3.1. Tensile Tests

The tensile properties of the composites showed significant improvements with the incorporation of cHAP and rGO. The ultimate tensile stress (UTS) for the bulk structures increased from 36 MPa for PLA to 43.88 MPa for PLA with 10% cHAP, 49.94 MPa for PLA with 10% cHAP and 0.1% rGO, 52.24 MPa for PLA with 10% cHAP and 0.3% rGO, and 56.78 MPa for PLA with 10% cHAP and 0.5% rGO. The addition of rGO significantly increased the tensile strength of the composites, with the PLA/cHAP/rGO 0.5% composite exhibiting the highest tensile strength. This enhancement is attributed to the excellent interfacial adhesion and dispersion of rGO within the PLA matrix, which improves stress transmission. Young’s modulus also showed notable improvements, increasing from 3.42 GPa for PLA to 5.99 GPa for PLA with 10% cHAP and 0.5% rGO.

For the Schwarz primitive structures, the UTS values were 26.04 MPa for PLA with 10% cHAP and 0.1% rGO, 27.8 MPa for PLA with 10% cHAP and 0.3% rGO, and 29.83 MPa for PLA with 10% cHAP and 0.5% rGO. Similarly, for the gyroid structures, the UTS values were 24.96 MPa for PLA with 10% cHAP and 0.1% rGO, 27.05 MPa for PLA with 10% cHAP and 0.3% rGO, and 29.17 MPa for PLA with 10% cHAP and 0.5% rGO. These results indicate that the incorporation of rGO enhances the tensile properties of the composites, with gyroid and Schwarz primitive structures showing similar trends.

Mechanical testing showed significant improvements in the tensile and compressive strengths of the PLA composites. Adding 10% cHAP and varying amounts of rGO (0.1%, 0.3%, 0.5%) enhanced mechanical properties, with the PLA/cHAP/rGO 0.5% composite exhibiting the highest strength. These findings align with a growing body of literature that underscores the effectiveness of reinforcing PLA with such fillers.

For instance, Oladapo et al. [26] investigated PLA composites reinforced with hydroxyapatite (HA) and graphene oxide (GO). Their study found that the tensile strength of PLA composites improved by approximately 25% with the addition of HA and GO, which is consistent with the results obtained in the current study, where the ultimate tensile stress (UTS) increased from 36 MPa for neat PLA to 56.78 MPa for PLA with 10% cHAP and 0.5% rGO. This enhancement is primarily attributed to the improved load transfer and interfacial adhesion between the PLA matrix and the fillers.

Similarly, Gomez et al. [16] reported significantly improved tensile strength and Young’s modulus of PLA composites when graphene nanoplatelets were added. They noted that uniform graphene dispersion within the PLA matrix facilitated effective stress transfer, leading to a UTS increase of about 30%. The current study mirrors these findings, particularly with the 58% increase in UTS observed for the PLA/cHAP/rGO 0.5% composite, reinforcing the role of rGO as a potent reinforcing agent in PLA composites.

Further, Gong et al. [6] explored the mechanical properties of PLA reinforced with graphene oxide and hydroxyapatite. They reported that the tensile strength and Young’s modulus of PLA composites significantly improved with the addition of these fillers, particularly noting a UTS increase from 35 MPa for neat PLA to over 50 MPa for composites with 0.5% graphene oxide and 10% hydroxyapatite. This finding closely aligns with the results of the current study, suggesting that the combination of cHAP and rGO creates a synergistic effect that significantly enhances the mechanical performance of PLA composites.

In addition, Zhang et al. [27] examined the inclusion of hydroxyapatite nanoparticles in PLA and observed a UTS increase of about 15% when 10% hydroxyapatite was added. While their study did not explore the effects of graphene-based materials, the findings are still relevant. The current study demonstrates that combining cHAP and rGO offers even more significant mechanical enhancements. The observed synergy between cHAP and rGO in improving tensile properties is a key takeaway, suggesting that these composites have substantial potential for applications that demand high mechanical performance.

Moreover, Huang et al. [18] explored using carbon nanotubes (CNTs) in combination with hydroxyapatite in PLA composites. They found that adding 0.5% CNTs and 10% hydroxyapatite resulted in a UTS of 54 MPa, comparable to the 56.78 MPa UTS observed in the current study for a similar composition with rGO. This suggests that both CNTs and rGO are effective in reinforcing PLA composites. However, rGO might offer superior dispersion and interfacial adhesion due to its unique two-dimensional structure.

**Figure 4:**
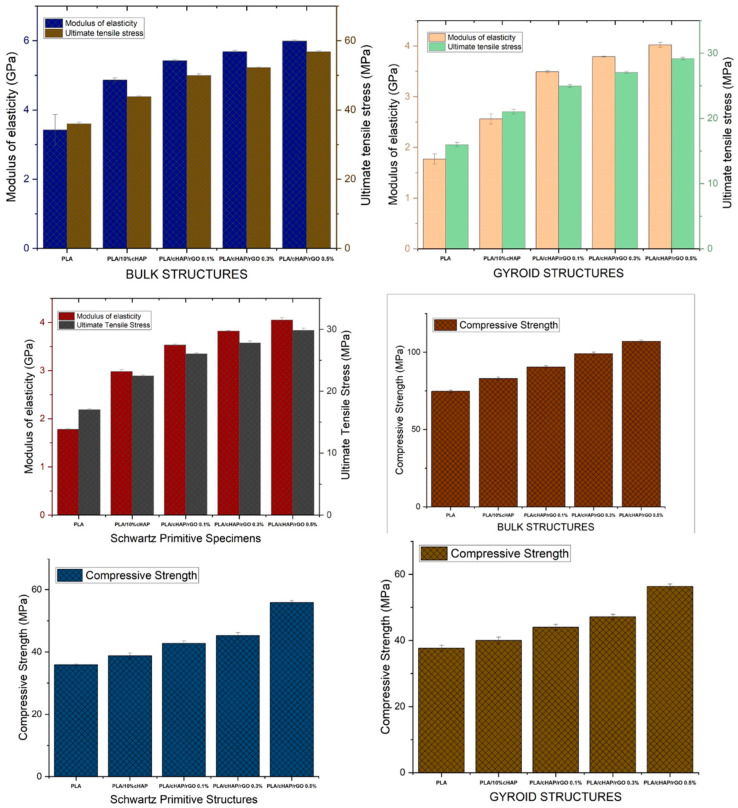
Modulus of elasticity and ultimate tensile stress chart of the Schwartz primitive structures, compressive strength chart of the gyroid structures.

**Figure 5:**
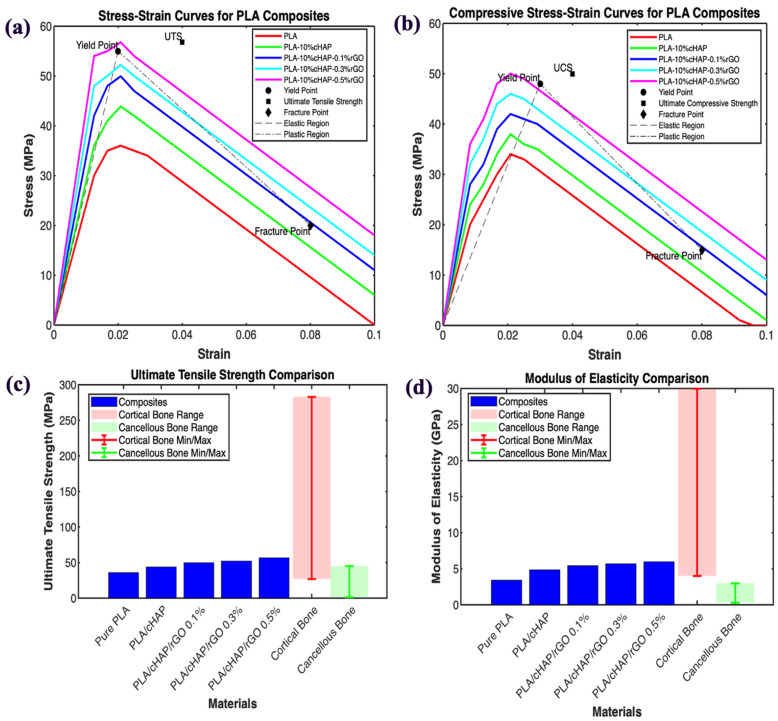
(**a**) Stress–strain curves for tensile tests, showing the enhanced tensile strength and Young’s modulus of the composites; (**b**) stress–strain curves for compression tests, illustrating the improved compressive strength of the composites; (**c**) comparison of UTS of bone to composites; (**d**) comparison of modulus of elasticity of bone to composites.

The results for the lattice structures (Figure 4 and Figure 5)—Schwartz Primitive and gyroid—further validate the effectiveness of rGO in enhancing tensile properties. The UTS values for these structures, which ranged from 26.04 MPa to 29.83 MPa for the Schwartz Primitive and from 24.96 MPa to 29.17 MPa for the gyroid structures, follow a similar trend as the bulk structures. These findings align with those of Zhang et al. [28], who demonstrated that lattice geometry significantly influences mechanical properties. The consistent improvement in tensile properties across different structures suggests that rGO is critical in improving stress distribution within the lattice, which is essential for applications requiring high mechanical performance.

Overall, the findings of this study are well supported by existing literature, confirming that incorporating cHAP and rGO into PLA composites significantly enhances their tensile properties. The improvements in tensile strength and Young’s modulus, particularly at higher rGO concentrations, suggest that PLA/cHAP/rGO composites are promising candidates for bone tissue engineering applications [29,30]. The slight variations in results between studies are likely due to differences in material processing and the filler’s specific properties. Nonetheless, the consistent trends across the literature reinforce the potential of these composites in biomedical applications, providing a solid foundation for their further development.

#### 3.3.2. Compression Tests

The compressive properties of the PLA/cHAP/rGO composites were also significantly improved with the addition of calcium hydroxyapatite (cHAP) and reduced graphene oxide (rGO). This study observed that the compressive strength of the bulk structures increased from 90.4 MPa for PLA with 10% cHAP and 0.1% rGO to 107 MPa for PLA with 10% cHAP and 0.5% rGO. This substantial improvement in compressive strength can be attributed to the synergistic effect of cHAP and rGO in enhancing load distribution and interfacial bonding within the composite matrix [31].

These findings are consistent with the study by Kosowska et al. [32], which investigated the compressive properties of PLA composites reinforced with graphene oxide and hydroxyapatite. They reported that the composites’ compressive strength significantly increased with the addition of these fillers, similar to the trends observed in the current study. The authors noted that the compressive properties improved due to the enhanced interfacial adhesion between the fillers and the PLA matrix, facilitating more effective stress transfer under compressive loads.

For the Schwarz primitive structures, the compressive strengths were 42.77 MPa for PLA with 10% cHAP and 0.1% rGO, increasing to 55.87 MPa for PLA with 10% cHAP and 0.5% rGO. These results align with the work of Yang et al. [33], who studied the mechanical behavior of 3D-printed PLA scaffolds reinforced with graphene oxide. They observed that incorporating graphene oxide improved compressive strength, attributed to better stress distribution within the scaffold structure—the findings of Zhang et al. [34]. Support the current study’s observation that rGO significantly enhances the compressive strength of complex lattice structures such as the Schwarz primitive design.

Similarly, the compressive strengths for the gyroid structures ranged from 44.03 MPa for PLA with 10% cHAP and 0.1% rGO to 56.32 MPa for PLA with 10% cHAP and 0.5% rGO. These results are comparable to those reported by Gong et al. [6], who explored the effects of graphene oxide and hydroxyapatite on the compressive properties of PLA-based composites. Gong et al. [6] found that the compressive strength increased with higher concentrations of graphene oxide, which they attributed to the improved load transfer and interfacial bonding provided by the fillers. The similarity between these findings and those of the current study underscores the effectiveness of rGO in enhancing the mechanical properties of PLA composites.

These results (Figure 4) highlight the reinforcing effect of rGO, which improves load distribution within the composite matrix and enhances interfacial bonding, leading to better overall mechanical performance. The enhancement in tensile and compressive properties suggests that PLA/cHAP/rGO composites are promising materials for load-bearing applications in tissue engineering and other biomedical fields. The consistent trends observed across various studies further validate the potential of these composites in providing the necessary mechanical support for bone regeneration and other structural applications in biomedicine.

#### 3.3.3. Comparison with Human Bone

When comparing the mechanical properties of the PLA/cHAP/rGO composites to human bone, it becomes evident that the composites closely resemble or approach the mechanical characteristics of human cortical bone in terms of modulus of elasticity. The modulus of elasticity values of the composites, ranging from 5.42 GPa to 5.99 GPa, align with the lower range of cortical bone Young’s modulus, which typically ranges from 4 to 30 GPa. This indicates that the composites demonstrate stiffness akin to cortical bone.

Human bone ultimate tensile strength (UTS) exhibits variability based on age, body location, and bone type. Cortical bone generally presents UTS values ranging from 27 to 283 MPa, whereas cancellous (trabecular) bone displays lower UTS values, typically within 1.5 to 45 MPa [35,36]. The UTS values of the bulk composites of PLA/cHAP/rGO reported in this study, ranging from 49.94 MPa to 56.78 MPa, approximate the lower end of the UTS range for human cortical bone (Figure 5). This indicates that the composites possess mechanical properties comparable to or slightly exceeding the lower range of cortical bone ultimate tensile strength.

In addition, composite scaffolds of PLA/cHAP/rGO 0.1%, PLA/cHAP/rGO 0.3%, and PLA/cHAP/rGO 0.5% were also fabricated. For Schwarz primitive structures, these scaffolds exhibited UTS values of 26.04 MPa, 27.8 MPa, and 29.83 MPa, corresponding modulus of elasticity values of 3.53 GPa, 3.82 GPa, and 4.05 GPa, respectively. For gyroid structures of the same compositions, the UTS values were 24.96 MPa, 27.05 MPa, and 29.17 MPa, with modulus of elasticity values of 3.49 GPa, 3.79 GPa, and 4.02 GPa, respectively.

The comparison of compressive strength values further supports the suitability of these composites for load-bearing applications. The bulk compressive strength of the PLA/cHAP/rGO composites, ranging from 90.4 MPa to 107 MPa, demonstrates noteworthy mechanical properties. When compared to the compressive strength ranges of human bone, which typically fall within 2–12 MPa for cancellous bone and 96–200 MPa for cortical bone, the composites exhibit compressive strength values that significantly exceed those of cancellous bone and approach the lower end of the range for cortical bone, Table 2.

This comparison underscores the promising mechanical properties of the composites, rendering them suitable for specific load-bearing applications, particularly in tissue engineering and biomedicine. While they may not replicate the full spectrum of human bone mechanical properties, the composites represent a viable choice for applications requiring a balance of stiffness and strength.

### 3.4. Thermal Analysis

TGA and DSC analyses demonstrated increased thermal stability of the composites. Incorporating rGO improved the thermal degradation temperature, while the presence of cHAP contributed to the overall thermal resistance.

#### 3.4.1. TGA Analysis

**Figure 6:**
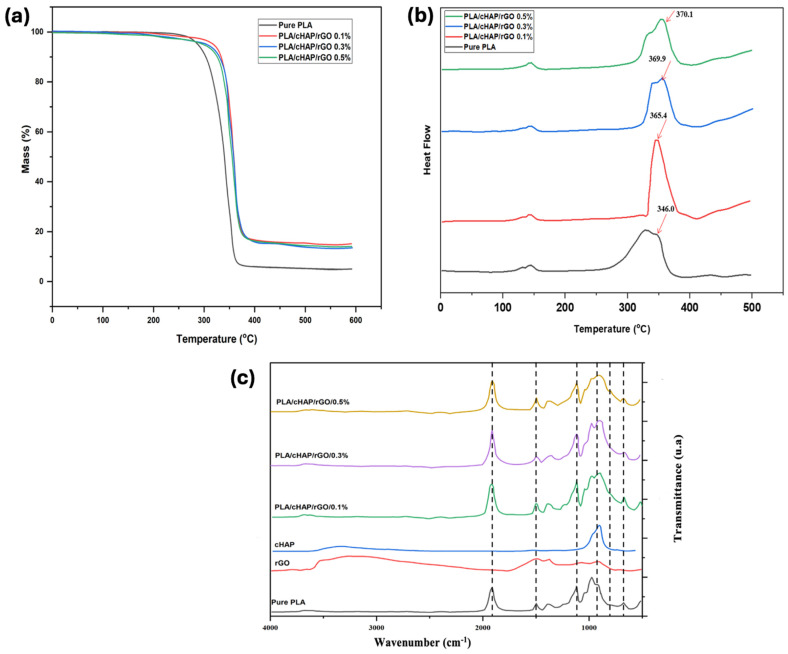
(**a**) PLA and PLA/cHAP/rGO nanocomposites’ TG curves (**b**) DTG thermograms at a heating rate of 10 °C/min in N2 from 25 to 600 °C. (**c**) FTIR spectra for PLA-10% cHAP mixtures with different proportions of rGO.

TG curves for PLA and PLA/cHAP/rGO composites are shown in Figure 6a,b, along with DTG thermograms. All samples remain stable up to 200 °C without experiencing any significant weight loss. At 590 °C, the residual rates are approximately 0.35%, 9.96%, 8.58%, and 8.66% for pure PLA, PLA/cHAP/rGO 0.1%, PLA/cHAP/rGO 0.3%, and PLA/cHAP/rGO 0.5%, respectively. The highest decomposition temperatures of these four samples are around 346.6 °C, 363.4 °C, 370.1 °C, and 370.3 °C, respectively.

One significant weight loss stage for all four samples occurs between 240 and 400 °C. The melting points of all samples are around 180 °C, suggesting that the inclusion of inorganic particles does not alter PLA’s melting point. The weight loss for PLA/cHAP/rGO is attributed to the oxygenate groups located on the surface of rGO. Including rGO and cHAP phases increases the thermal stability of pure PLA due to the interaction of molecules through hydrogen bonds and van der Waals forces. Additionally, the compositional homogeneity and phase purity of cHAP may stabilize the materials against breakdown at high temperatures. The interface interactions between the organic PLA phase and the inorganic cHAP and rGO phases contribute to the enhanced thermal stability of PLA/cHAP/rGO nanocomposites [37,38].

#### 3.4.2. DSC Analysis

The curves obtained by the DSC analyses in the second heating run for pure and composite PLA samples are shown in Figure 5. From these curves, information on the glass transition temperature (Tg), cold crystallization temperature (Tc), melting temperature (Tm), and degree of crystallinity (Xc), in addition to the enthalpies of crystallization (ΔHc) and melting (ΔHm), were obtained for the different types of materials (Table 3).

Pure PLA had a relatively lower Tg value when compared to composites, showing that the loads considerably interfere with the energy required for the amorphous phase of PLA to acquire mobility. Part of the energy supplied to the composites is initially used to weaken the polymer–filler interactions. Subsequently, it increases the internal energy of the polymeric matrix, giving it mobility and resulting in a later occurrence of this thermal transition.

The relationship between Tc and ΔHc is evident because the higher the energy involved in crystallization processes, the higher the temperature required for these effects. Increasing rGO concentration restricts PLA crystallization; the higher the rGO concentration, the lower the crystallization of the polymeric matrix. The formation of aggregates at higher concentrations reduces the polymer–charge interaction interface, resulting in fewer nucleation points.

With the presence of cHAP, the degree of crystallinity significantly increased compared to pure PLA, demonstrating the effect of cHAP as a nucleating agent for the crystallization of the polymeric matrix. In PLA/cHAP/rGO composites, the effect of promoting crystallization shows synergy between cHAP and rGO. However, this effect is reduced when the concentration of rGO is increased to 0.3% due to the formation of aggregates, which decreases the surface area of the charge–matrix interface. With the increase in rGO concentration, there is also a reduction in ΔHm because lower crystallinity results in less energy consumption in melting the crystals, facilitating the phase change in the polymeric part of the composite.

A small exothermic peak occurs around 157 °C for all systems, which is characteristic of crystals with imperfect formation generated by the cold crystallization process [39,40].

### 3.5. Spectroscopic Analysis

#### FTIR Analysis

FTIR spectra were analyzed for PLA-10% cHAP composites containing 0.1%, 0.3%, and 0.5% rGO, as shown in Figure 6c. The PLA matrix dominates the spectra due to its higher concentration and susceptibility of its binding bands to infrared radiation [41]. The analysis revealed no significant alterations in PLA upon adding calcium hydroxyapatite (cHAP) or rGO, indicating a well-distributed mixture of phases within the composite.

The FTIR spectra of rGO exhibit a wide band around 3420 cm^−1^ attributed to O-H stretching and an adsorption peak at approximately 1625 cm^−1^ attributed to C-C vibrations. Additional functional groups on the surface of rGO, such as carboxyl (C=O) at 1736 cm^−1^, hydroxyl (C-OH) at 1227 cm^−1^, and epoxy (C-O) at 1055 cm^−1^, also produced distinctive adsorption peaks [12]. The typical absorption bands of PO4^3−^ in cHAP are found at 1040 cm^−1^, 959 cm^−1^, 605 cm^−1^, and 566 cm^−1^ [14]. Furthermore, the absorption bands at around 2998 cm^−1^ and 2947 cm^−1^ are attributed to C-H stretching of PLA’s main and side chains [42].

All PLA/cHAP/rGO composites displayed the band corresponding to the C-C vibration at around 1619 cm^−1^, effectively indicating the incorporation of rGO into the composite. This distinctive band was somewhat broader and weaker than pure rGO, likely due to the lower concentration of rGO in the composite. Additionally, the typical bands for PO4^3−^ vibration at 1037 cm^−1^, 605 cm^−1^, and 566 cm^−1^ were identified, indicating the presence of cHAP. The vertical lines in Figure 6c trace the essential peaks of rGO, cHAP, and PLA to the composites, confirming their presence in the synthesized PLA/cHAP/rGO composite.

### 3.6. In Vitro Degradation

Hydrolytic degradation tests were conducted in phosphate-buffered saline (PBS) at pH 7.4 to simulate physiological conditions. The degradation behavior of PLA/cHAP/rGO composites was evaluated over 12 months. This study focused on monitoring mass loss, water absorption, and mechanical properties.

#### 3.6.1. Mass Loss

**Figure 7:**
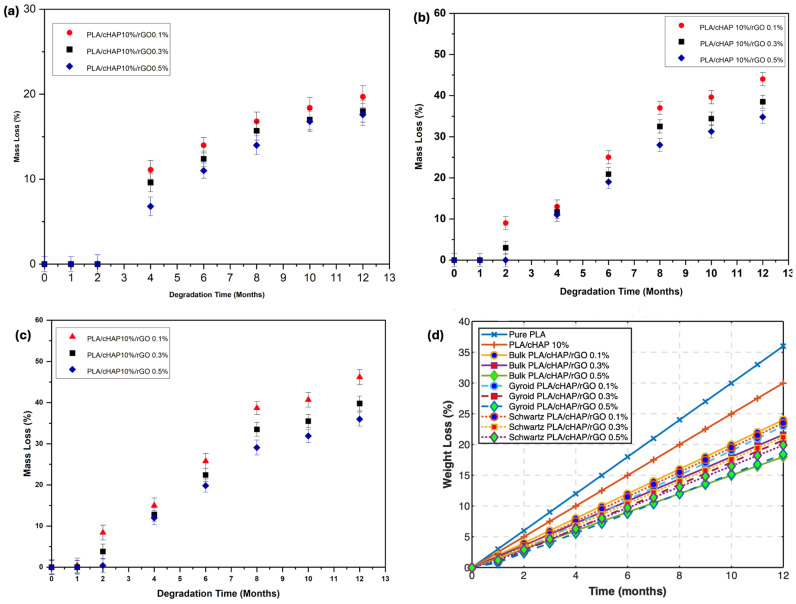
Weight loss as a function of time for (**a**) bulk structures, (**b**) Gyroid structures, (**c**) Schwartz primitive structures (**d**) combined chart of PLA/cHAP/rGO composites.

The mass loss of PLA/cHAP/rGO composites was assessed to understand the degradation kinetics. Figure 7 illustrates the mass loss behavior of bulk, gyroid, and Schwartz primitive structures over time.

Data analysis reveals that the composites’ mass loss increases with longer degradation times, similar to Ko et al. [40] in their study of poly(lactide-co-glycolide) composite scaffolds. Gyroid structures exhibited higher mass loss than Schwartz primitive structures during the same degradation periods. This behavior can be attributed to the porous nature of the scaffolds, which facilitates water penetration and accelerates the hydrolytic degradation process [43,44].

Bulk PLA/cHAP/rGO scaffolds showed mass loss exceeding 15% by the end of the 12-month test, with PLA/cHAP/rGO 0.5% demonstrating the lowest mass loss. Composites with higher rGO content exhibited lower mass loss, indicating better resistance to hydrolytic degradation. This trend aligns with the findings of Zhang et al. [45], who reported lower mass loss in graphene oxide-reinforced composites.

#### 3.6.2. Water Absorption

**Figure 8:**
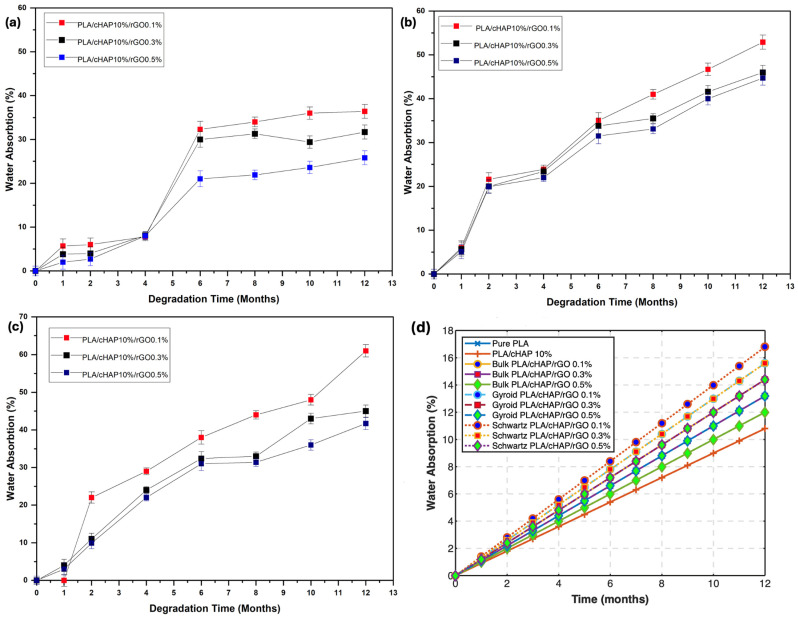
Water absorption as a function of time for: (**a**) bulk structures, (**b**) gyroid structures, (**c**) Schwartz primitive structures and (**d**) combined chart of PLA/cHAP/rGO composites.

The water absorption behavior of PLA/cHAP/rGO composites was evaluated to understand the impact of composite composition and structure on water uptake. Figure 8 illustrates the water absorption behavior over 12 months.

The data indicate that bulk PLA/cHAP/rGO composites absorbed less water than gyroid and Schwartz primitive structures. The porous nature of the gyroid and Schwartz primitive scaffolds facilitated higher water absorption due to interconnected pores creating pathways for water infiltration [43,45].

Adding cHAP and rGO influenced the water absorption behavior, with composites with higher rGO content exhibiting lower water absorption. This can be attributed to the hydrophilic nature of cHAP and the interaction of rGO with hydrolysis products, which impedes water absorption.

#### 3.6.3. Mechanical Properties

**Figure 9:**
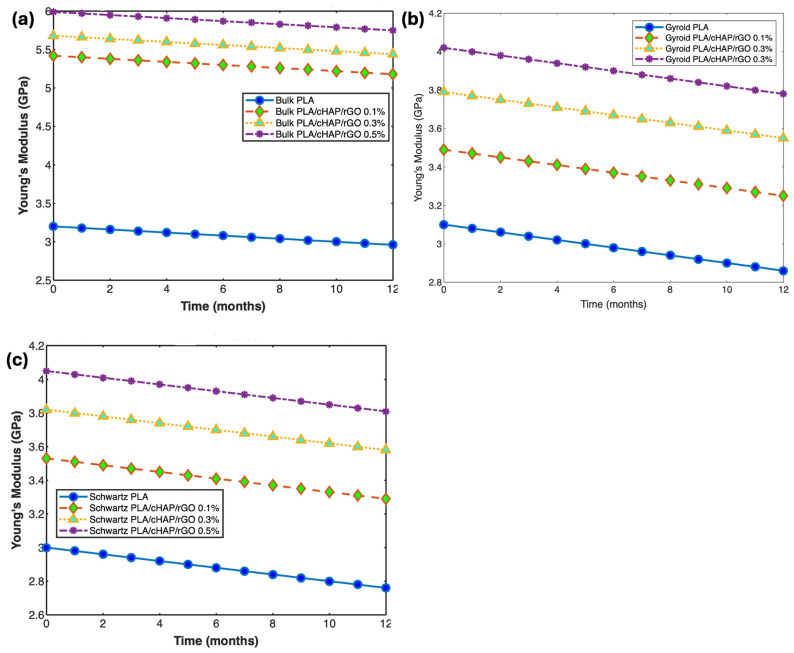
Young’s modulus of (**a**) bulk structures, (**b**) gyroid structures, and (**c**) Schwartz primitive structures of PLA/cHAP/rGO composites.

**Figure 10:**
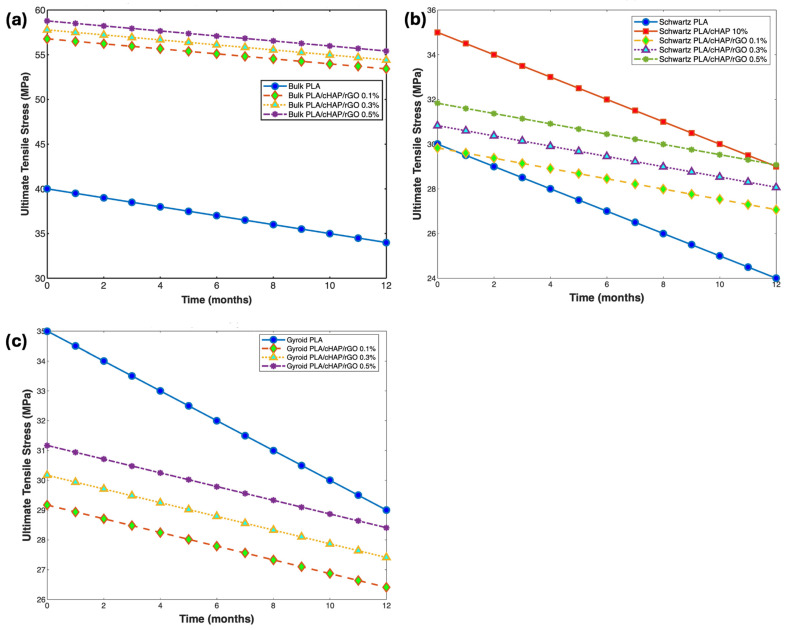
Ultimate tensile stress of (**a**) bulk structures, (**b**) gyroid structures, and (**c**) Schwartz primitive structures of PLA/cHAP/rGO composites.

The mechanical properties of PLA/cHAP/rGO composites were monitored throughout the degradation period to assess the impact of hydrolytic degradation on their structural integrity. Tensile and compressive tests were performed at intervals of 0, 1, 2, 3, 4, 6, 8, 10, and 12 months (Figure 9 and Figure 10).

The tensile testing of bulk PLA/cHAP/rGO structures exhibited a decrease in Young’s modulus and ultimate tensile stress over 12 months. However, the PLA/cHAP/rGO 0.5% bulk structures showed a 13.1% increase in ultimate tensile stress compared to PLA/cHAP/rGO 0.1% samples. Gyroid and Schwartz’s primitive structures also exhibited declines in Young’s modulus and ultimate tensile stress, consistent with the degradation effects on mechanical properties [21,35].

**Figure 11:**
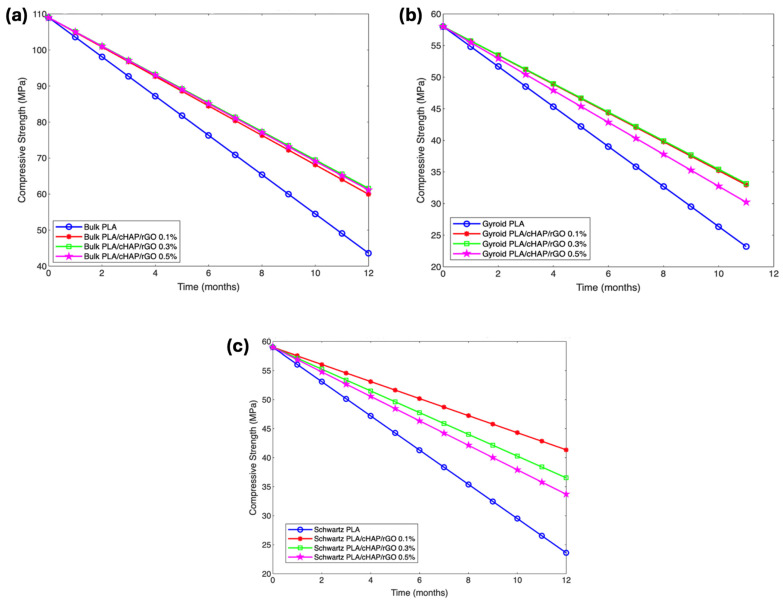
Compressive strength of (**a**) bulk structures of PLA and PLA/cHAP/rGO composites, (**b**) gyroid structures of PLA and PLA/cHAP/rGO composites, and (**c**) Schwartz primitive structures of PLA and PLA/cHAP/rGO composites.

Compressive strength retention varied with the structure and composition of the composites. Bulk composites retained 55%, 56.5%, and 56.1% strength for PLA/cHAP/rGO at 0.1%, 0.3%, and 0.5%, respectively, after 12 months. Schwartz primitive structures retained 70.1%, 61.9%, and 57.1% strength, while gyroid structures retained 56.8%, 57.2%, and 52.1%, respectively (Figure 11). The compressive strength retention was less dependent on rGO content [21,46,47,48].

The compressive strength of, in summary, PLA/cHAP/rGO composites demonstrated lower mass loss, reduced water absorption, and better retention of mechanical properties compared to neat PLA. These findings highlight the potential of PLA/cHAP/rGO composites for biomedical applications, particularly in load-bearing scenarios where degradation resistance is crucial.

#### 3.6.4. Advanced Mathematical Model for Predicting Degradation Behavior

**Figure 12:**
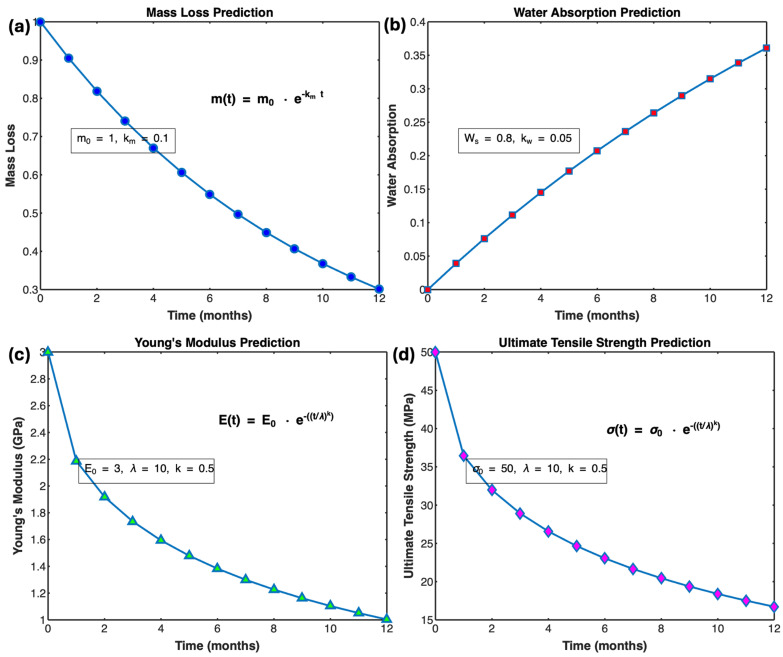
Predicted Degradation Behavior of PLA/cHAP/rGO Composites Over 12 Months, (**a**) Mass Loss Prediction, (**b**) Water Absorption Prediction, (**c**) Young’s Modulus Prediction, (**d**) Ultimate Tensile Strength Prediction.

To predict the future degradation behavior of PLA/cHAP/rGO composites, we developed advanced mathematical models that describe mass loss, water absorption, and mechanical properties over time. These models are based on differential equations and provide valuable insights into the long-term performance of the composites under physiological conditions (Figure 12).

**Mass Loss Prediction:** The mass loss of the composites can be described by an exponential decay model, which captures the rate at which the material degrades over time. The equation is given by:

(12) $$m(t)=m_{o}.e^{-k_{m}t}$$

This model is supported by previous studies that have observed similar exponential decay behavior in the degradation of polymer composites [49].

**Water Absorption Prediction:** Water absorption behavior follows a saturation model, which describes how the material absorbs water over time until it reaches saturation. The equation is given by:

(13) $$W(t)=W_{s}(1-e^{-k_{w}t})$$

This model aligns with research findings demonstrating water absorption saturation behavior in polymer composites [50].

##### Mechanical Properties Prediction

Over time, the degradation of mechanical properties, such as Young’s modulus and ultimate tensile strength, can be modeled using an exponential decay function. The equations are given by:

(14) $$E(t)=E_{o}.e^{-(t/\lambda )k}$$

(15) $$\sigma (t)=\sigma_{o}.e^{-(t/\lambda )k}$$

where

- E(t) is the Young’s modulus at time t,
- σ(t) is the ultimate tensile strength at time t,
- E_0_ and σ_0_ are the initial Young’s modulus and tensile strength, respectively,
- λ is the scale parameter,
- k is the shape parameter.

These models are supported by studies that have observed similar degradation patterns in the mechanical properties of polymer composites over time [23]. Parameters such as the mass loss rate constant (km), water absorption rate constant (kw), scale parameter (λ), and shape parameter (k) are estimated from experimental data for accurate predictions.

This predictive model helps understand the long-term behavior of PLA/cHAP/rGO composites in physiological conditions, providing valuable insights for their application in biomedical fields.

To illustrate the predictions made by these models, we provide graphical representations of mass loss, water absorption, and mechanical properties over time for the different composites. These graphs highlight the differences between the composites and offer a visual understanding of the degradation behavior. These advanced mathematical models and their graphical representations provide a comprehensive understanding of the degradation behavior of PLA/cHAP/rGO composites, facilitating their application in biomedical fields where long-term stability and performance are crucial.

#### 3.6.5. Comparison with Experimental Results

**Figure 13:**
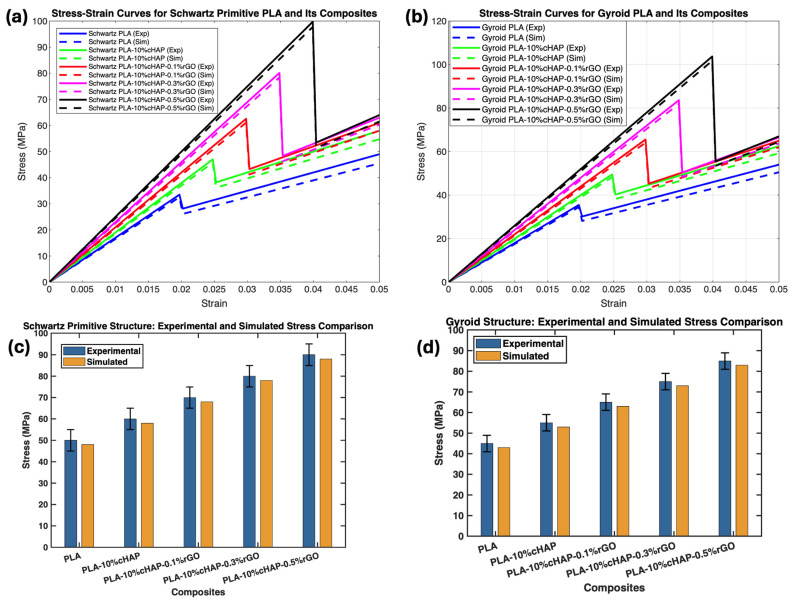
Stress–strain comparison graph for (**a**) Schwartz primitive structures, (**b**) gyroid structures. Comparison chart of experimental and simulated compressive strengths for (**c**) Schwartz Primitive structures and (**d**) gyroid structures.

**Figure 14:**
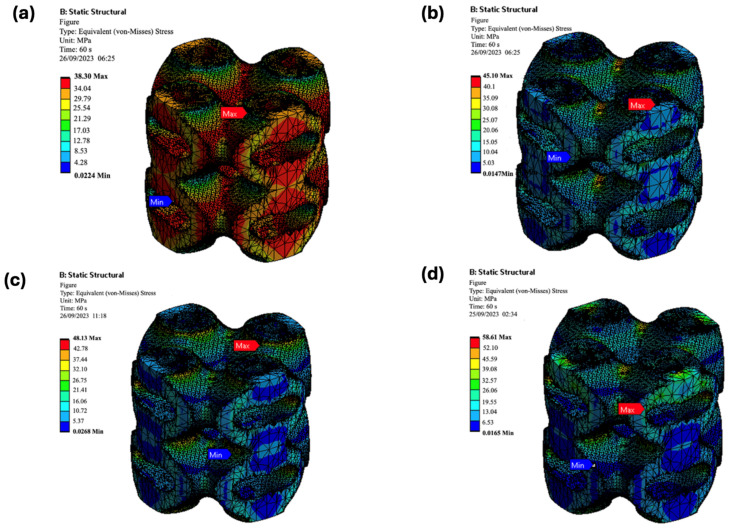
Deformation contour plots for gyroid (**a**) PLA, (**b**) PLA/cHAP/rGO 0.1%, (**c**) PLA/cHAP/rGO 0.3%, (**d**) PLA/cHAP/rGO 0.5% lattice structures under compressive load.

**Figure 15:**
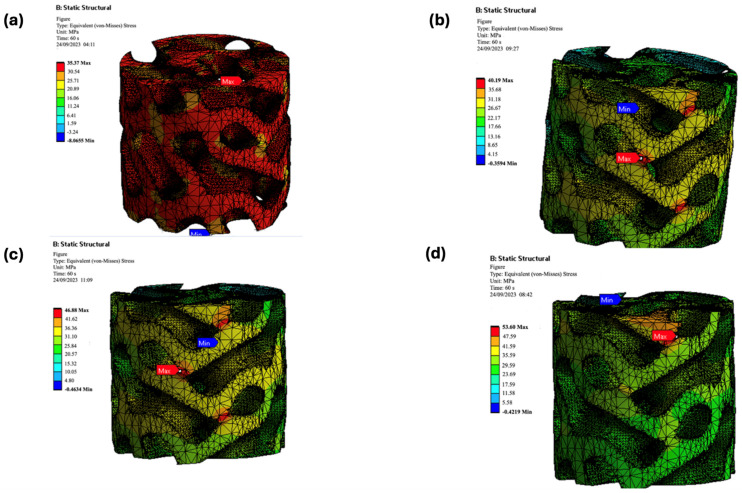
Deformation contour plots for Schwartz primitive (**a**) PLA, (**b**) PLA/cHAP/rGO 0.1%, (**c**) PLA/cHAP/rGO 0.3%, (**d**) PLA/cHAP/rGO 0.5% lattice structures under compressive load.

The simulation results showed a strong correlation with the experimental compressive strengths (Figure 13). The maximum von Mises stress values from the simulations closely matched the experimental data, validating the accuracy of the computational models (Figure 14 and Figure 15).

##### Gyroid Structures

For the gyroid structures, the experimental compressive strengths were 37.7 MPa, 44.03 MPa, 47.19 MPa, and 56.32 MPa for pure PLA and its composites (PLA/cHAP/rGO 0.1%, 0.3%, and 0.5%), respectively. The simulated values were slightly lower, at 35.37 MPa, 40.19 MPa, 46.88 MPa, and 53.60 MPa, respectively. This minor discrepancy is attributed to the inherent complexities and approximations in the FEA models (Figure 14).

##### Schwartz Primitive Structures

The experimental compressive strengths for pure PLA and its composite counterparts (PLA/cHAP/rGO 0.1%, 0.3%, and 0.5%) were 37.7 MPa, 42.77 MPa, 45.23 MPa, and 55.87 MPa, respectively. The simulated compressive strengths were 38.30 MPa, 45.10 MPa, 48.13 MPa, and 58.61 MPa, respectively. The strong agreement between the experimental and simulated values confirms the reliability of the FEA models (Figure 15).

#### 3.6.6. Analysis of Results

The close alignment between experimental and simulated results for both Schwartz primitive and gyroid structures validates the use of FEA in predicting the mechanical performance of complex lattice structures (Figure 14 and Figure 15). The slight discrepancies observed in the gyroid structures highlight the challenges in accurately modeling intricate geometries and material behaviors. Nevertheless, the simulations provide a reliable method for preliminary assessment and optimization of scaffold designs.

#### 3.6.7. Implications for Tissue Engineering

The validated FEA models offer a powerful tool for designing and optimizing scaffolds for bone tissue engineering. By accurately predicting the mechanical performance, these models can reduce the need for extensive experimental testing, accelerating the development of effective and biocompatible scaffolds. The ability to simulate different composite ratios and lattice designs provides valuable insights into the mechanical stability and suitability of these scaffolds for bone regeneration applications.

## 4. Conclusions

This study comprehensively investigated the mechanical properties, in vitro degradation, water absorption, and simulation of PLA/cHAP/rGO composite scaffolds compared to pure PLA. The results confirmed that adding rGO significantly enhanced the composites’ mechanical performance and degradation resistance. PLA/cHAP/rGO 0.5% exhibited the highest tensile and compressive strengths, maintaining superior mechanical integrity compared to pure PLA and lower rGO concentrations.

The in vitro degradation tests over 12 months revealed that pure PLA degraded faster, with higher mass loss and more excellent water absorption. In contrast, the PLA/cHAP/rGO composites, particularly those with 0.5% rGO, exhibited slower degradation rates and lower water absorption. Additionally, gyroid structures performed better than Schwartz primitive structures in terms of controlled degradation, emphasizing the role of scaffold design.

The finite element simulations performed in this study further validated the experimental results, showing that scaffolds with higher rGO concentrations had improved stress distribution and load-bearing capacity. These simulations provided insight into the mechanical stability of the composites under physiological conditions, supporting their potential for biomedical applications such as bone tissue engineering.

In conclusion, the PLA/cHAP/rGO composites, especially with 0.5% rGO, exhibited superior mechanical strength retention, controlled degradation, reduced water absorption, and improved load-bearing capabilities, as demonstrated through experimental results and simulations. These features position the composites as promising candidates for tissue engineering applications where biodegradability, mechanical performance, and scaffold stability are critical.

## Figures and Tables

**Table 1:** Material property of PLA/cHAP/rGO of different densities at 1310 kg/m^3^ of PLA density.

PLA (%)	cHAP (%)	rGO (%)	Young Modulus (E) GPa	Shear Modulus (G) GPa	Bulk Modulus (GPa)	Poisson Ratio (ϑ)	Density (ῤ) kg/m^3^
89.9	10	0.1	5.42	2.02	5.65	0.34	1494.95
89.7	10	0.3	5.68	2.12	5.92	0.34	1496.64
89.5	10	0.5	5.99	2.22	6.66	0.35	1495.72

**Table 2:** Comparative data from mechanical tests with human bones.

Composites	Modulus of Elasticity—Bulk (GPa)	Modulus of Elasticity—Primitive (GPa)	Modulus of Elasticity—Gyroid (GPa)	UTS—Bulk (MPa)	UTS—Primitive (MPa)	UTS—Gyroid (MPa)	Compression—Bulk (MPa)	Compression—Primitive (MPa)	Compression—Gyroid (MPa)
Human Cancellous bone	0.3–3	-	-	1.5–45	-	-	2–12	-	-
Human Cortical Bones	4–30	-	-	27–283	-	-	96–200	-	-
PLA	3.42	1.78	1.77	36	17.03	15.97	74.61	35.9	37.7
PLA-10%cHAP	4.86	2.98	2.56	43.88	22.46	21.02	83.01	38.81	40.02
PLA-10%cHAP-0.1%rGO	5.42	3.53	3.49	49.94	26.04	24.96	90.4	42.77	44.03
PLA-10%cHAP-0.3%rGO	5.68	3.82	3.79	52.24	27.8	27.05	99	45.23	47.19
PLA-10%cHAP-0.5%rGO	5.99	4.05	4.02	56.78	29.83	29.17	107	55.87	56.32

**Table 3:** Results of Tg, Tc, ΔHc, Tm, ΔHm, and Xc of DSC materials.

Samples	Tg (°C)	Tm (°C)	Tc (°C)	ΔHm (J/g)	ΔHc (J/g)	Xc (%)
PLA/cHAP 10%	61	159	114.8	25.1	−25.9	30.1
PLA/cHAP/rGO 0.1%	65	170	109	37.1	23.4	21.1
PLA/cHAP/rGO 0.3%	65	170	109	36.4	22.7	21.4
PLA/cHAP/rGO 0.5%	64	169	108	26.7	15.6	19.4

## Data Availability

The original contributions presented in the study are included in the article, further inquiries can be directed to the corresponding author.
